# A routine blood parameter–based nomogram to predict prolonged fever in children with primary EBV infection

**DOI:** 10.3389/fped.2026.1840358

**Published:** 2026-07-21

**Authors:** Ying Ding, Chunyu Lu, Kun Wang, Dandan Jin, Jiahui Wu, Jianmei Tian, Fangfang Cheng

**Affiliations:** Department of Infectious Diseases, Children's Hospital of Soochow University, Suzhou, China

**Keywords:** Epstein–Barr virus infections, fever, nomogram, prediction model, routine blood parameters

## Abstract

**Objective:**

This study aimed to develop a clinical nomogram using routine blood parameters to identify the risk of prolonged fever (>1 week) in children with acute primary Epstein–Barr virus (EBV) infection.

**Methods:**

This retrospective study involved 791 hospitalized children with primary EBV infection. Patients were divided into two groups: fever duration ≤1 week and >1 week. Candidate routine blood parameters were screened through univariate analysis. Least Absolute Shrinkage and Selection Operator (LASSO) regression was used for variable selection to mitigate overfitting. A multivariate logistic regression model was then constructed using the optimal predictors and visualized as a nomogram. Model performance was rigorously quantified through the area under the receiver operating characteristic curve (AUC), calibration plots, and decision curve analysis (DCA), with internal validation performed using 1,000 bootstrap resamples.

**Results:**

Prolonged fever affected 27.4% of the cohort, and antiviral therapy was administered to all these patients (100%), significantly higher than the 56.1% in the short fever group (*P* < 0.001). LASSO regression identified white blood cell count (WBC), neutrophil count (NEU), platelet count (PLT), and atypical lymphocyte proportion (AL) as optimal predictors. The nomogram including these four parameters predicted prolonged fever with an AUC of 0.728 (95% CI: 0.689–0.768). Calibration and DCA confirmed the nomogram's clinical utility.

**Conclusion:**

A nomogram based on four routine blood parameters (WBC, NEU, PLT and AL) provides a practical method for the early risk stratification of children at high risk for prolonged EBV-related fever. This model serves as an initial reference, though its clinical value requires validation through multicenter prospective studies.

## Introduction

Epstein–Barr virus (EBV) is a ubiquitous γ-herpesvirus affecting over 95% of the adult population worldwide (1). Primary infection typically occurs during childhood. EBV infection in immunocompetent children elicits a vigorous cellular immune response characterized by pronounced proliferation of activated cytotoxic CD8+ T lymphocytes that target and clear infected B cells (2). This immunopathological process manifests as the classic triad of infectious mononucleosis: fever, tonsillopharyngitis, and cervical lymphadenopathy (3). Most children with EBV infection have a self-limiting clinical course, yet some develop fever that persists for more than one week (4). Prolonged fever in pediatric EBV infection increases distress and anxiety for patients and families. Identifying children prone to a protracted course is key to managing expectations and guiding observation, but simple predictive tools are currently lacking.

Complete blood count is a fundamental, cost-effective, and rapidly accessible diagnostic tool in clinical practice. Routine blood parameters, including direct measures such as white blood cell count (WBC), platelet count (PLT) and hemoglobin (HGB), as well as derived parameters such as the neutrophil-to-lymphocyte ratio (NLR), platelet-to-lymphocyte ratio (PLR) and systemic immune-inflammation index (SII), have been shown to possess significant value in risk stratification and prognostic assessment of various pediatric infectious diseases (5–7). Additionally, in EBV infection, the proportion of atypical lymphocytes (AL) is an important parameter reflecting the degree of cytotoxic T-cell activation (8).

This study aimed to determine whether routinely available blood parameters could predict prolonged fever in immunocompetent children with primary EBV infection. Using least absolute shrinkage and selection operator (LASSO) regression and multivariate logistic regression, we developed a nomogram and assessed its discrimination, calibration and clinical utility. Our objective was to evaluate whether these parameters could provide preliminary guidance for predicting fever duration, potentially aiding in the initial assessment of clinical course and helping clinicians manage parental expectations in children with primary EBV infection.

## Materials and Methods

### Study population

This single-center retrospective study enrolled children with primary EBV infection who were hospitalized at Children's Hospital of Soochow University between January 2019 and December 2023. This study was conducted in accordance with the principles of the Declaration of Helsinki and was approved by the Medical Ethics Committee of Children's Hospital of Soochow University (Approval No. 2025CS175). Given the retrospective design, the requirement for written informed consent was waived.

### Inclusion and exclusion criteria

Inclusion criteria were as follows: (1) age ≤16 years; (2) Presenting with more than three clinical manifestations consistent with EBV infection, including fever, tonsillopharyngitis, cervical lymphadenopathy, splenomegaly, hepatomegaly, and periorbital edema; (3) at least one of the following laboratory criteria confirming primary EBV infection: positive Epstein–Barr virus capsid antigen immunoglobulin M (EBV VCA-IgM) and positive Epstein–Barr virus capsid antigen immunoglobulin G (EBV VCA-IgG) with negative Epstein–Barr virus nuclear antigen immunoglobulin G (EBNA-IgG); or negative EBV VCA-IgM but positive EBV VCA-IgG with low-avidity antibodies. EBV VCA-IgG avidity was determined using a commercial chemiluminescence immunoassay, with low avidity defined as an avidity index <0.40 according to the manufacturer's cut-off.

Patients were excluded if they had any of the following: (1) immunodeficiency (primary or secondary) or use of immunosuppressive agents; (2) confirmed co-infection with other pathogens (bacterial, viral, or fungal); (3) underlying diseases affecting white blood cell, platelet, or hemoglobin counts; (4) severe EBV-related complications, including hemophagocytic lymphohistiocytosis (HLH), EBV encephalitis, meningitis, or immune thrombocytopenic purpura (ITP); (5) incomplete clinical or laboratory data.

**Figure 1:**
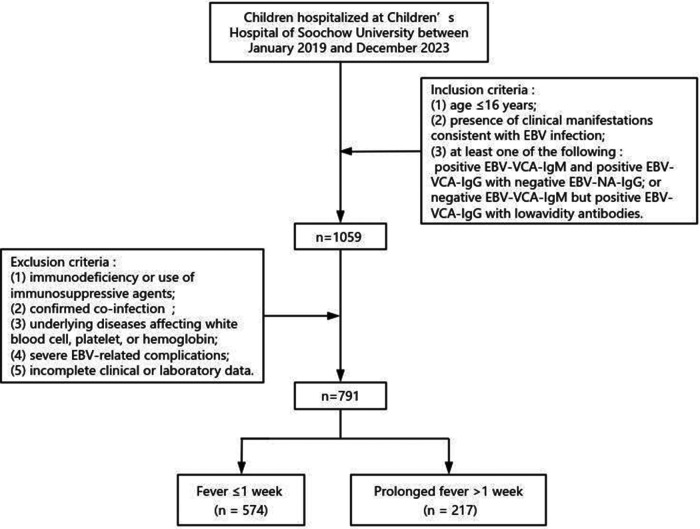
Flow chart of study subject selection. A total of 791 immunocompetent children with primary EBV infection were included in the final analysis after rigorous application of inclusion and exclusion criteria. EBV, Epstein–Barr virus.

The patient selection and exclusion process is illustrated in Figure 1. The sample size was determined by the availability of eligible cases during the study period.

### Data collection

This study collected data on patient baseline characteristics (age, gender, days before admission), routine blood parameters, duration of fever and antiviral therapy. Routine blood parameters were collected from outpatient results on the day of admission or from tests performed within 24 h of hospitalization. The following peripheral blood parameters were assessed: WBC, HGB, PLT, neutrophil (NEU), lymphocyte (LYM) and AL. Based on these, the following derived inflammatory indices were calculated: PLR = platelet count/lymphocyte count (×10^9^/L), NLR = neutrophil count/lymphocyte count (×10^9^/L), SII = platelet count × neutrophil count/lymphocyte count (×10^9^/L). Antiviral therapy with acyclovir or ganciclovir, administered either orally or intravenously, was included in the analysis. Two researchers independently extracted and cross-checked all data.

### Statistical analyses

Statistical analysis was conducted using R software (version 4.5). Non-normally distributed continuous variables were expressed as medians (Q1, Q3) and compared using the Mann–Whitney *U* test. Categorical variables were presented as frequencies (%) and compared by *χ*^2^ test or Fisher's exact test as appropriate. Univariate analysis compared the following parameters between groups: WBC, HGB, PLT, NEU, LYM, PLR, NLR, SII, AL. Variables with *P* < 0.10 (a threshold chosen to avoid excluding potentially important predictors) were entered into LASSO regression for optimal predictor selection. Multivariate binary logistic regression identified factors independently associated with prolonged fever and a nomogram was constructed using the rms package. Variance inflation factors (VIFs) were calculated to check for multicollinearity among predictors, using a cutoff of <5. Model performance was evaluated by calibration (Hosmer–Lemeshow test), discrimination (AUC) and clinical utility [decision curve analysis (DCA) and clinical impact curve (CIC)]. All tests were two-sided, *P* < 0.05 was considered significant.

## Results

### Univariate analysis of baseline characteristics between the two groups

A total of 791 immunocompetent children hospitalized with primary EBV infection were ultimately included in this study. Patients were stratified into two groups based on fever duration: 574 (72.6%) in the ≤1 week group and 217 (27.4%) in the prolonged fever (>1 week) group. No significant differences were observed in age, gender, or days before admission between the groups (all *P* > 0.05). The prolonged fever group had significantly higher levels of WBC, PLT, NEU, LYM, NLR, SII and AL compared to the ≤1 week group (all *P* < 0.05), while HGB and PLR showed no significant differences between the groups. The proportion of patients receiving antiviral therapy was significantly higher in the prolonged fever group (217/217, 100%) than in the short fever group (322/574, 56.1%; *P* *<* 0.001). The above data are presented in Table 1.

**Table 1:** Baseline characteristics and routine blood parameters of immunocompetent children with primary EBV infection.

Items	Short fever group (*N* = 574)	Prolonged fever group (*N* = 217)	Statistic	*P*
age in months	60.50 (41.00, 81.00)	58.00 (42.00, 80.00)	0.526	0.599
Male/Female	310/264	125/92	0.823	0.364
Days before admission	4.00 (3.00, 5.00)	4.00 (3.00, 6.00)	−0.845	0.398
WBC (10^9^/L)	13.16 (10.88, 16.03)	14.41 (11.87, 18.76)	−4.351	<0.001
HGB (g/L)	119.06 (114.00, 126.00)	119.09 (116.00, 125.00)	−0.510	0.610
PLT (10^9^/L)	204.00 (164.00, 225.00)	224.00 (196.00, 265.00)	−6.468	<0.001
NEU (10^9^/L)	2.91 (2.12, 3.50)	3.45 (2.81, 4.39)	−7.089	<0.001
LYM (10^9^/L)	8.76 (6.91, 11.38)	9.45 (7.50, 12.18)	−1.977	0.048
PLR	22.23 (16.11, 30.18)	23.73 (17.36, 31.86)	−1.896	0.058
NLR	0.32 (0.22, 0.46)	0.36 (0.27, 0.50)	−3.724	<0.001
SII	59.70 (38.05, 95.65)	83.09 (55.47, 119.08)	−6.150	<0.001
AL (%)	5.00 (3.00, 6.00)	6.00 (4.00, 8.00)	−4.022	<0.001
antiviral use	322	217	139.7	<0.001

Data are presented as median (Q1, Q3) or number. WBC, white blood cell count; HGB, hemoglobin; PLT, platelet count; NEU, neutrophil count; LYM, lymphocyte count; PLR, platelet-to-lymphocyte ratio; NLR, neutrophil-to-lymphocyte ratio; SII, systemic immune-inflammation index; AL, atypical lymphocyte proportion.

### LASSO regression analysis for variable selection

**Figure 2:**
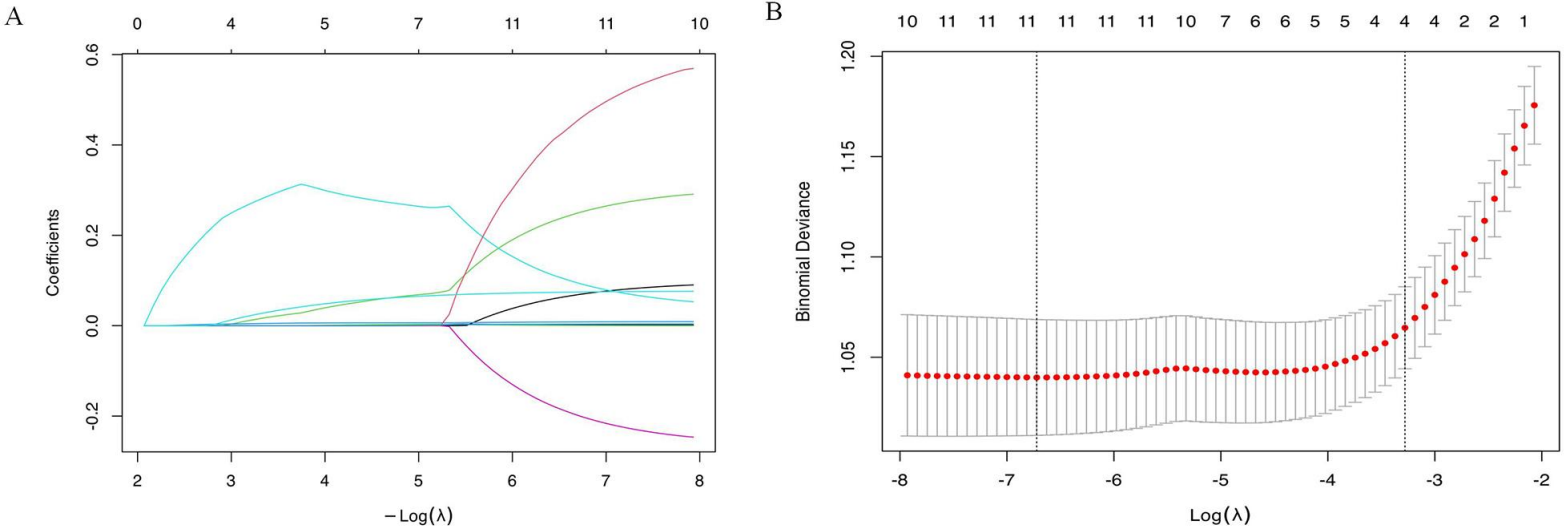
Variable selection using LASSO regression from candidate routine blood parameters. **(A)** Coefficient profile plot showing the shrinkage of candidate variable coefficients as the penalty parameter λ increases; **(B)** Cross-validation binomial deviance curve; the optimal λ was selected using the one-standard-error criterion (indicated by the right vertical dashed line), resulting in the retention of four key predictors: WBC, NEU, PLT, and AL. WBC, white blood cell count; NEU, neutrophil count; PLT, platelet count; AL, atypical lymphocyte proportion.

To identify independent predictors, variables with *P* < 0.10 in univariate analysis (WBC, PLT, NEU, LYM, PLR, NLR, SII and AL), together with sex and age in months, were entered as candidate variables. To address potential multicollinearity, LASSO regression was used to penalize coefficients. As shown in Figure 2, the one-standard-error criterion from cross-validation yielded a parsimonious model with optimal predictive performance. Finally, four variables retained non-zero coefficients: WBC, PLT, NEU and AL.

### Multivariate binary logistic regression analysis

The four variables were entered into multivariate binary logistic regression analysis. All four variables emerged as independent risk factors for fever lasting longer than one week, with odds ratios >1 and *P* < 0.05. The results are presented in Table 2. Multicollinearity diagnostics revealed no significant collinearity among the four predictors, all VIFs were well below the threshold of 5 (WBC: 3.24, NEU: 3.87, PLT: 1.12, and AL: 1.08). Based on these findings, a nomogram was constructed to visualize the predictive model.

**Table 2:** Multivariate binary logistic regression analysis identifying routine blood parameters independently associated with prolonged fever (>1 week) in pediatric primary EBV infection.

Variable	beta	SE	Wald *χ*^2^	*P*	OR (95%CI)
(Intercept)	−5.269	0.503	109.503	<0.001	
WBC (10^9^/L)	0.054	0.019	7.781	0.005	1.056 (1.016–1.097)
PLT (10^9^/L)	0.008	0.002	29.484	<0.001	1.008 (1.005–1.011)
NEU (10^9^/L)	0.39	0.074	27.937	<0.001	1.477 (1.278–1.706)
AL (%)	0.074	0.02	14.027	<0.001	1.077 (1.036–1.119)

WBC, white blood cell count; NEU, neutrophil count; PLT, platelet count; AL, atypical lymphocyte proportion.

### Development and performance evaluation of the nomogram prediction model

**Figure 3:**
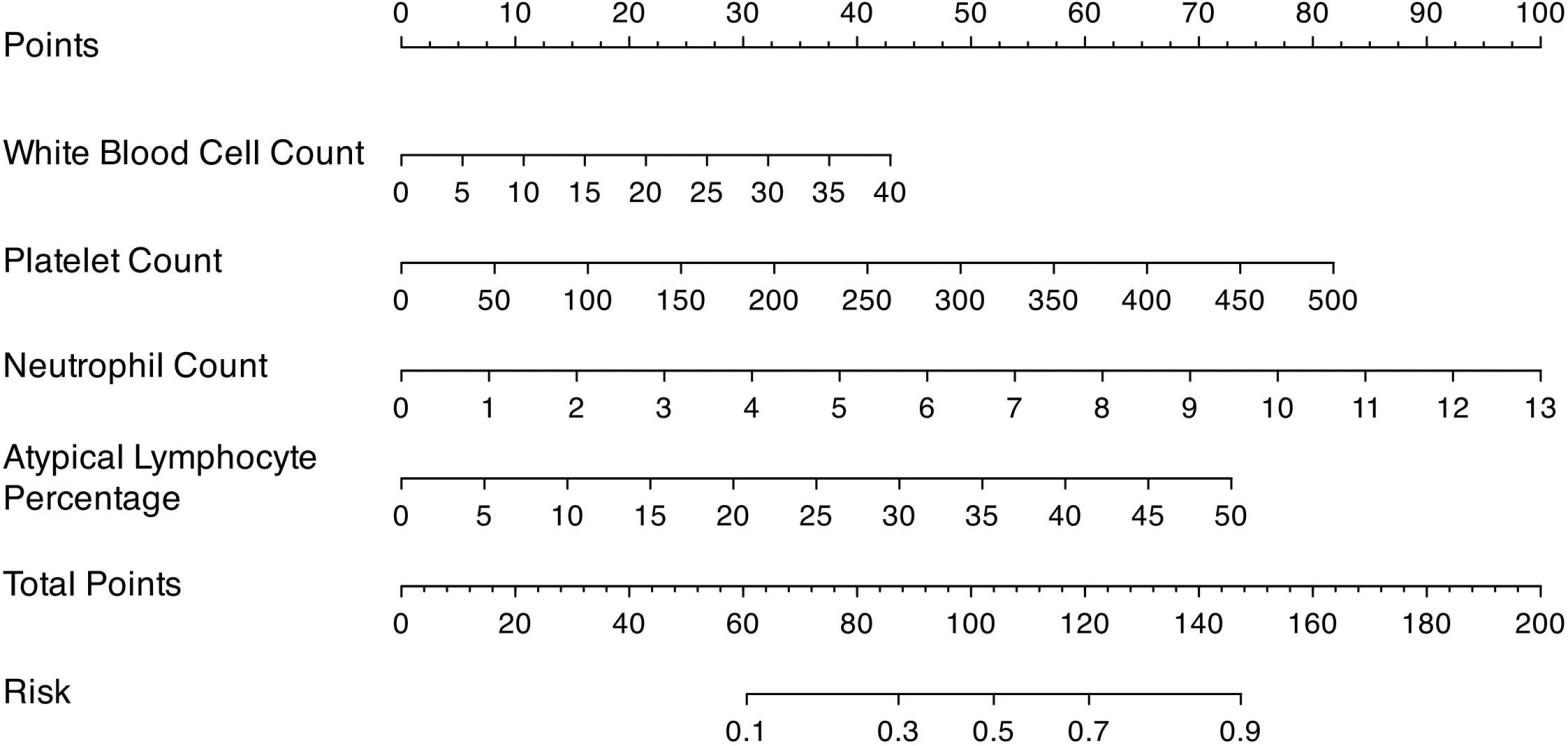
Nomogram for predicting the risk of prolonged fever in immunocompetent children with primary EBV infection. The total score, calculated by summing the points assigned to the four parameters (WBC, NEU, PLT, and AL), corresponds to the predicted probability of fever duration >1 week. EBV, Epstein–Barr virus; WBC, white blood cell count; NEU, neutrophil count; PLT, platelet count; AL, atypical lymphocyte proportion.

**Figure 4:**
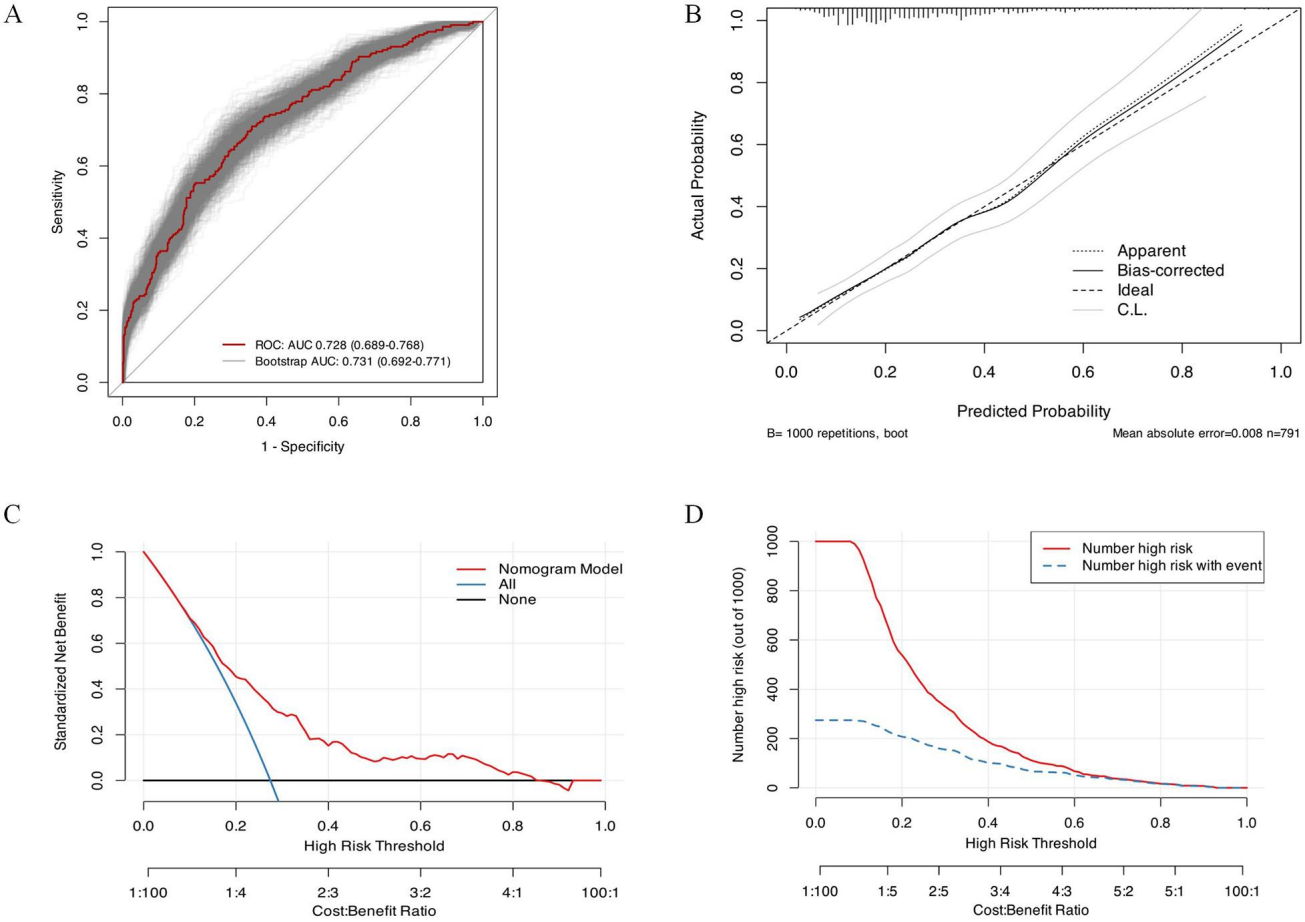
Performance evaluation of the nomogram prediction model. **(A)** ROC curve showing an AUC of 0.728 (95% CI: 0.689–0.768); **(B)** Calibration curve of the nomogram after 1,000 bootstrap resamples, demonstrating good agreement between the predicted and observed probabilities of prolonged fever (mean absolute error = 0.008); **(C)** DCA indicating positive net benefit across threshold probabilities of 9%–85%. **(D)** CIC showing effective identification of high-risk cases within the 20%–30% threshold range. ROC, receiver operating characteristic; AUC, area under the receiver operating characteristic curve; CI, confidence interval; DCA, decision curve analysis; CIC, clinical impact curve.

The nomogram (WBC, PLT, NEU and AL; Figure 3) showed an AUC of 0.728 (95% CI: 0.689–0.768) for predicting prolonged fever, with an optimism-corrected AUC of 0.731 (95% CI: 0.692–0.771) via 1,000 bootstrap resamples (Figure 4A). The Hosmer–Lemeshow test yielded *χ*^2^ = 5.534 (*P* = 0.699). The calibration curve, plotted with 1,000 bootstrap resamples, demonstrated good agreement between predicted and observed probabilities, with a mean absolute error of 0.008 (Figure 4B). DCA showed that the nomogram provided a positive net benefit across threshold probabilities ranging from 9% to 85% (Figure 4C). CIC demonstrated high predictive accuracy with low false-positive rates, particularly within the 20%–30% threshold range (Figure 4D).

## Discussion

Primary EBV infection is a common infectious disease in children. The infection may involve multiple organs, including the liver, spleen, lungs, brain and bone marrow. Fever is the most frequent clinical presentation. Its duration is closely associated with disease severity and the risk of complications (9). In this study, we developed and internally validated a nomogram to predict prolonged fever. In several previous retrospective studies of children with primary EBV infection, the median duration of fever was approximately 7 days (4, 9, 10), therefore, prolonged fever in this study was defined as fever lasting more than 7 days. The model comprises four routine blood parameters: WBC, PLT, NEU and AL. A total of 791 immunocompetent children were included, among whom 217 (27.4%) experienced prolonged fever. The two groups were comparable in terms of age and gender. The median time from symptom onset to admission was 4 days in both groups (*P* = 0.398). *Post hoc* sample size verification confirmed adequacy, with an events-per-variable ratio of 54.3 for the four predictors, well above the recommended minimum of 10 events per variable for logistic regression and also satisfying the more stringent criterion of up to 50 events per variable suggested when variable selection is performed (11). The nomogram achieved moderate predictive performance with an AUC of 0.728, good calibration and favorable clinical utility across a range of threshold probabilities. Because all four parameters are routinely measured on complete blood count, the nomogram offers a practical tool for early risk stratification.

In this study, the prolonged fever group showed significantly higher levels of WBC, NEU, LYM and AL compared to the short fever group. This observation is consistent with the established immunopathological features of EBV infection. Primary EBV infection initially targets B cells, followed by a strong cellular immune response characterized by clonal expansion and activation of CD8+ T cells (2). Recent studies show extensive changes in total and subset WBC during acute EBV infection (12). Notably, neutrophils with increased CD64 expression are significantly elevated (13). EBV uses its envelope glycoprotein gp350 to bind neutrophils, promoting antiviral cytokines like interleukin-1α (IL-1α) and interleukin-1β (IL-1β) (14). Activated neutrophils release chemokines to shape the local inflammatory microenvironment and may modulate adaptive immunity via T cells (15). The elevated WBC and NEU in the prolonged fever group likely reflects a strong inflammatory response to EBV, which may be a potential driver for prolonged fever. Multicollinearity diagnostics confirmed that despite the biological correlation between these parameters, no significant collinearity was present (all VIFs <5), supporting the stability of the multivariable model.

Although lymphocyte counts differed significantly between the two groups, this variable was not selected in the final LASSO regression model. LASSO regression handles multicollinearity by shrinking coefficients and tends to retain the most representative predictors when multiple variables carry similar information (16). In this analysis, the predictive information from lymphocyte counts may have been captured by the AL. AL directly reflect the proportion of activated CD8+ T cells, the lymphocyte subset most relevant to EBV-specific immune responses (8). EBV infection induces large numbers of activated cytotoxic T lymphocytes. These cells are essential for eliminating infected B cells, yet their activation also drives immunopathology through the release of inflammatory cytokines (17). This process is directly reflected by the proportion of atypical lymphocytes in peripheral blood.

Platelets have emerged as key immunoregulators, far beyond their traditional roles as simple hemostatic agents (18). In this study, the significantly elevated platelet count in the prolonged fever group may reflect inflammation-driven reactive thrombocytosis. EBV infection alters platelet transcriptomes and functions (19). Activated platelets bridge innate and adaptive immunity. They upregulate surface P-selectin to form platelet-neutrophil aggregates via P-selectin glycoprotein ligand-1 (PSGL-1), promoting neutrophil activation (20), and release CD40L to recruit and activate T lymphocytes (21). However, one study has shown that EBV-DNA load is negatively correlated with platelet count in children with primary EBV infection (22). This suggests that changes in platelet count may reflect different pathophysiological stages of EBV infection: an early reactive increase may indicate inflammatory activation, while a later decrease due to consumption may signal disease progression. Overall, the precise mechanisms underlying the role of platelets in EBV infection warrant further investigation.

The inclusion of PLT together with WBC, NEU and AL suggests that these four indicators capture different aspects of the immune-inflammatory response in EBV infection. WBC and NEU represent the intensity of innate immune activation. AL reflects the strength of the adaptive immune response. PLT may indicate platelet-mediated immune regulation and inflammatory crosstalk. This multidimensional combination may explain the model's clinical net benefit on DCA, despite its moderate predictive performance.

NLR, PLR and SII have demonstrated significant predictive value across various viral infectious diseases (23, 24). But in this study, PLR showed no significant difference between the two groups. This may be explained by the synchronous elevation of both PLT and L in the prolonged fever group, which offset their ratio and diminished the discriminative ability of PLR. Although NLR and SII differed significantly in univariate analysis, they were not selected in the LASSO regression model. This is due to the nature of LASSO regression in handling multicollinearity: when composite indices and their component variables enter the model together, LASSO tends to retain the more representative original variables. In children with EBV infection, the actual counts of WBC, NEU, PLT and AL may reflect immune activation better than their ratios do. For this reason, original blood parameters may be more clinically useful here than derived indices.

The 100% antiviral rate in the prolonged fever group reflects a reactive clinical strategy, in which clinicians initiate therapy in response to persistent fever. Although the difference in antiviral use between the two groups was significant, this variable was not included in the model as it represents a post-admission intervention. That marked difference, however, raises the possibility of confounding by indication or reverse causality: treatment decisions were inherently tied to the evolving illness course, so the observed prolonged fever does not represent a pure natural-history outcome. The clinical benefit of antiviral therapy in pediatric EBV infection remains uncertain (25, 26). This underscores the need for a risk stratification tool to assist clinical decisions, helping target treatment to those most likely to benefit while minimizing unnecessary exposure in low-risk children. Antiviral therapy should be guided not by empirical practice alone, but by a careful integration of factors including EBV DNA load and clinical presentation.

To our knowledge, this study appears to be the first to establish a predictive model for prolonged fever in pediatric primary EBV infection using only routine blood parameters. Despite the moderate AUC of 0.728, DCA confirmed its clinical utility for risk stratification. Across a wide threshold range of 9% to 85%, the model offered higher net benefit than monitor-all or monitor-none strategies. Within the 20% to 30% threshold range, it effectively identified children who actually developed prolonged fever, with a low false-positive rate. These findings suggest the model is useful as a preliminary tool for early assessment. The nomogram offers a practical way to evaluate potential fever duration based solely on routine blood tests. In resource-limited settings, where advanced testing is often unavailable, flagging children at higher risk can guide clinical observation and help clinicians manage family expectations, thereby reducing anxiety about the illness course.

This study has several limitations. First, it was a single-center retrospective study enrolling only hospitalized children, which may introduce selection bias. Second, the lack of data on pre-hospital antiviral use may confound the assessment of fever duration. Third, the current model did not include EBV DNA load or cytokine profiles. Although these tests may offer additional predictive value, their longer laboratory turnaround times limit routine use. Despite these limitations, the model offers preliminary practical value due to its simplicity and use of routinely available tests. Future multicenter prospective studies are needed to validate these findings. Researchers should also explore whether serial blood measurements or alternative parameter combinations could further improve predictive performance.

## Conclusions

This study developed a simple nomogram using routine blood parameters (WBC, NEU, PLT and AL) to predict prolonged fever in children with primary EBV infection. The model showed moderate predictive performance and offers a potential approach to risk stratification. By identifying low-risk children early, it may help clinicians adopt a more conservative observation strategy and manage parental expectations about the illness course. Future external validation is needed to confirm the generalizability of these findings across diverse pediatric populations.

## Data Availability

The raw data supporting the conclusions of this article will be made available by the authors, without undue reservation.
